# Targeted differential photostimulation alters reproductive activities of domestic birds

**DOI:** 10.3389/fphys.2022.1040015

**Published:** 2022-12-02

**Authors:** I. Rozenboim, J. Bartman, N. Avital Cohen, N. Mobarkey, S. Zaguri, M. E. El Halawani, Y. Chaiseha, A. Marco

**Affiliations:** ^1^ Department of Animal Sciences, Robert H. Smith Faculty of Agriculture, Food and Environment, Hebrew University of Jerusalem, Rehovot, Israel; ^2^ Department of Animal Science, University of Minnesota, Minneapolis, MN, United States; ^3^ School of Biology, Institute of Science, Suranaree University of Technology, Nakhon Ratchasima, Thailand

**Keywords:** broilers, photostimulation, extraretinal photoreceptors, reproduction, targeted illumination

## Abstract

Modern poultry production systems use environmentally controlled houses providing only artificial illumination. The role of light in reproduction of poultry depends on light quality (photoperiod, intensity/brightness, and spectrum), which enables us to provide custom-made illumination, targeted for the elevation of reproductive activities. Artificial targeted illumination significantly affects poultry reproduction. This phenomenon is based on the mechanism of light absorption in birds, which consists of two main components: the eye (retinal photoreceptors) and brain extraretinal photoreceptors. Several experiments on turkey hens and broiler breeder males and females have shown that photostimulation of brain extraretinal photoreceptors, while maintaining retinal photoreceptors under non-photostimulatory conditions, elevates reproductive activity by increasing egg production of hens and semen quality of roosters. In addition, we found acceleration in all gonadal axis parameters, leading to the acceleration in the production rate. Furthermore, we studied the role of retinal activation in gonadal axis suppuration and identified the role of serotonin in this phenomenon. As for today, several broiler breeder farms use targeted illumination based on our studies with excellent results.

## Introduction

**FIGURE 1:**
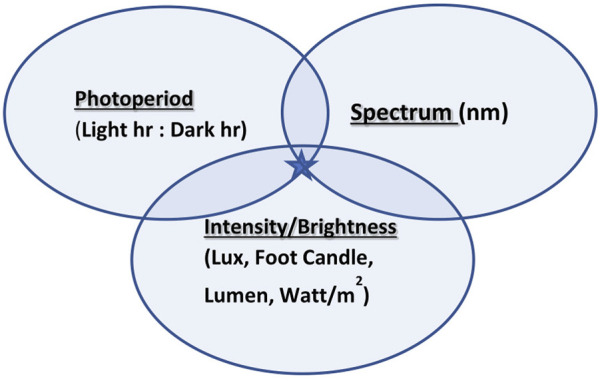
Three components of light quality: photoperiod, intensity/brightness, and spectrum join together to create targeted illumination (marked with a star).

In modern environmentally controlled poultry houses, artificial illumination is provided (Rozenboim et al., 2013). Light quality is defined and manifested by photoperiod, intensity/brightness, and spectrum (Figure 1) and plays a pivotal role as an environmental factor activating reproduction. Its effect on bird reproduction has been studied for many years in order to accelerate productivity. Photoperiod as a use for the diphenism of day length is a common tool for the activation or deactivation of reproductive activities (Lewis and Morris, 2006). The terms light intensity and light brightness are mistakenly mixed due to misunderstanding of light perception in avian species. The intensity of any electromagnetic radiation (visual light is a small part of electromagnetic radiation) is measured by watts/m^2^, while brightness (a unit that reflects the effect of light on retinal photoreceptors) is measured by lux (lx), foot candle, and lumen. The third component of light quality is the spectral output of the light source measured in nanometers (nm).

Light perception in most of the avian species occurs at two major sites: the eye through the retina (retinal photoreceptors) and several places in the brain by extra-retinal photoreceptors (ERPRs) (Menaker and Underwood, 1976). Several studies presented extraretinal locations that included the pineal gland, olfactory bulb, and hypothalamus (Scanes and Dridi, 2021) and were defined as deep brain photoreceptors. In mammalian species, where no ERPR can be found, the only place governing the circadian rhythm is retinal photoreceptors (Tosini et al., 2016). The avian retinal system is not required for controlling the circadian rhythm and circannual cycle, as shown in enucleated ducks, which responded to photostimulation, by activating reproduction (Benoit and Assenmacher, 1954). In addition, a follow-up study (Benoit, 1964) found that covering the head to eliminate light penetration to the skull resulted in photorefractoriness. The biochemistry of photoreceptors presents opsin–protein complexes that bind to vitamin-A—which isomerizes in response to light (Bownds, 1967; Hart, 2001). This phenomenon of isomerization allows the opsin molecule to bind to a protein involved in signal transfer to the brain—which by activating the biochemical cascade alters neurotransmitter release from the photoreceptor (Applebury and Hargrave, 1986).

Photostimulation initiates activity in several parts of the brain by activating neuroendocrine response in several axes and causes a broad cascade of hormonal changes. By activating the release of hypothalamic gonadotropin-releasing hormone (GnRH), followed by the secretion of gonadotropins (LH and FSH) from the pituitary gland into the blood, there is an initiation of gonadal recrudescence (Chaiseha and El Halawani, 2005). Moreover, photostimulation reduces the production of gonadotropin-inhibitory hormone (GnIH), which acts as an inhibitory neuropeptide regulating the production of GnRH (Zaguri et al., 2020). While acting as a catalyst for the activation of the gonadal axis, photostimulation can cause a decline in the activity of various neuroendocrine axes, such as lactotropic and serotonergic axes (El Halawani et al., 1983; Dawson et al., 2002). Photostimulation is associated with a decrease in serotonin, which creates a chain reaction lowering levels of VIP, prolactin, and melatonin (Mobarkey et al., 2010).

Several studies have indicated that the eyes play an inhibitory role in reproductive activities of birds. Siopes and Wilson (1980) demonstrated that the eye of the Japanese quail was not essential for photostimulation and sexual development. However, the eyes appear to be essential for short-day-induced testicular regression. Yokoyama and Farner (1976) demonstrated the inhibitory effect of retinal photoreceptors on reproduction of white-crowned sparrows, manifested by a reduction in LH serum. In addition, Homma et al. (1972) and Siopes and Wilson (1974) similarly demonstrated the debilitating role of eyes in the photosexual responses of quails by reduction until termination of egg production and deteriorating cloacal gland activity due to shortened photoperiods, which affected only the birds with retinal vision.

The decline in the egg production rate during the reproductive season of domestic birds is a well-known phenomenon. Many studies were conducted in order to reveal the factors associated with this natural decline in productivity. Both incubation behavior and photorefractoriness are associated with environmental light stimulation.

By using differential targeted photostimulation, that is, activating the ERPR while maintaining the retina under non-photostimulatory conditions, we were able, among others, to determine the role of the eye and the brain in the decline of reproductive activities. This was the main objective of several studies conducted on turkey hens and broiler breeder hens and roosters in our laboratory.

## Early observations on turkey hens

Our first study was conducted in collaboration with Prof. Mohamed El Halawani from the University of Minnesota. In this first trial, we tested our theory on turkey hens. In brief, 384 large white turkey hens aged 20 weeks were housed in three environmental- and light-controlled rooms (*n* = 128). In each room, birds were housed in 16 pens (*n* = 8). In this experiment, we used filtered light (Lee filters). Two parallel light systems were installed in two experimental rooms. The first system (red) had peak emission in the 650–725 nm range (0.565 W/cm^2^; 4.45 lx), and the second system (Green) had peak emission between 500 and 575 nm (0.248 W/cm^2^; 23.1 lx). Before photostimulation birds were kept under 6 h of light using both red and green light systems, photostimulation was conducted by increasing the day length to 16 h of light either using the red-light system (red group) or the green-light system (green group) and providing the light for another 6 hr at the middle of the day. The third group was photostimulated by white light (full spectrum provided using the 60-W incandescent lamp, 13.4 W/cm^2^; 33.7 lx).

We observed that hens photostimulated with the green light that was exposed also to a short day of the red wavelength showed very low egg production. Hens receiving the full spectrum of light showed slightly higher egg production, while birds photostimulated with the red light that were exposed to short green photostimulation showed the highest egg production. Total egg production for 27 weeks was higher in the red group, followed by the white group and the green-treated birds.

At the end of this preliminary trial, we found that rearing turkey hens under a long day of red light combined with a short day of green light caused significant acceleration in egg production. By using dual lighting systems, we were able to create two parallel photostimulation conditions. First, by using the red light, the extraretinal photoreceptors were photostimulated with little photostimulation of the retinal system, and second, by using the green light, the retinal photoreceptors were photostimulated with little photostimulation of the extraretinal photoreceptors. It is clear from our observations that photostimulation of the retina causes an inhibitory effect on reproductive activities of turkey hens, while photostimulation of the extraretinal photoreceptors accelerates reproductive activities.

## The effect of targeted photostimulation on broiler breeder hens

**FIGURE 2:**
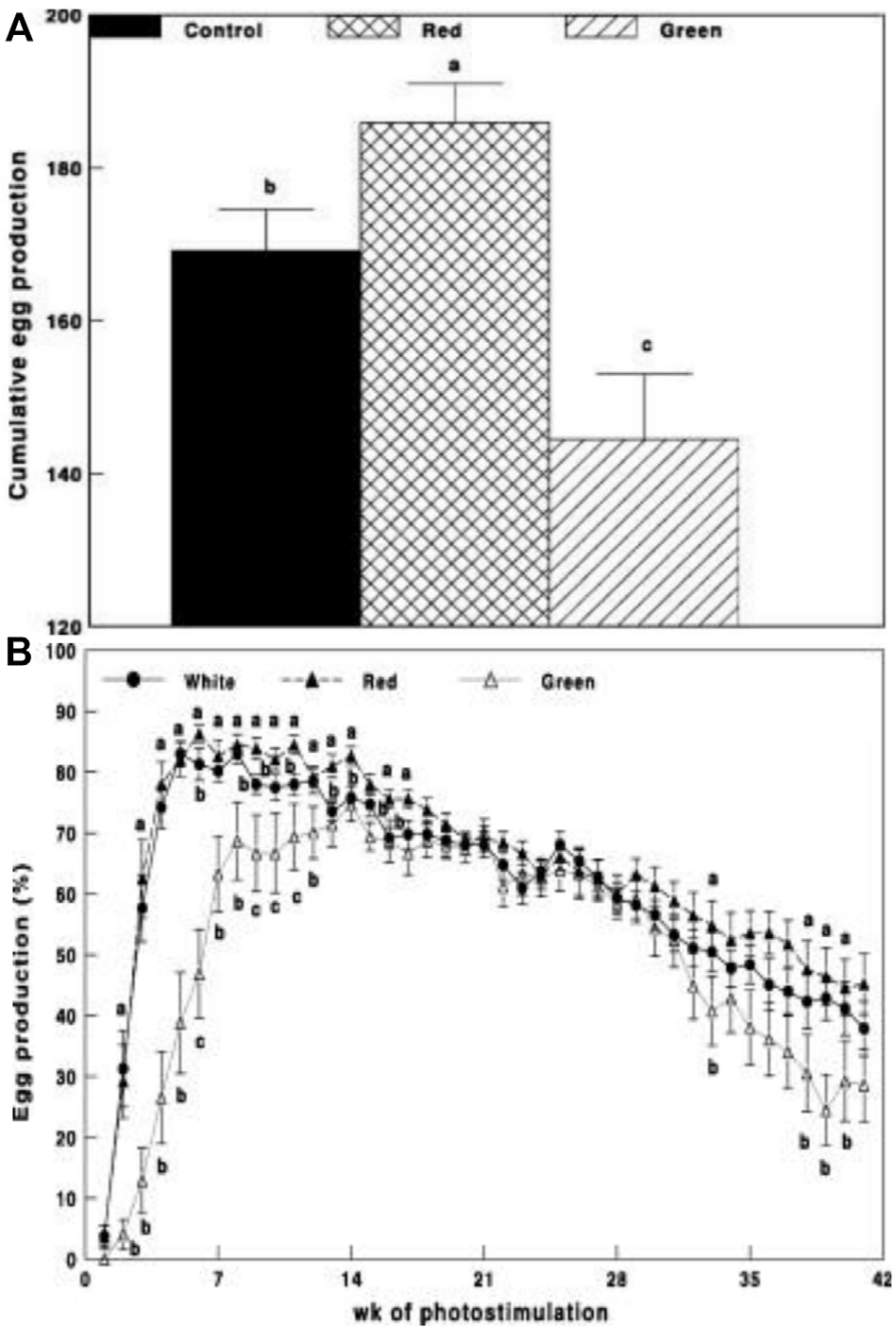
Cumulative egg production **(A)** and egg production through weeks of photostimulation (%) **(B)** of Cobb broiler breeder hens in the control group (29 lx, 0.1 W/m^2^); red group, photostimulated with the red light (0.1 W/m^2^) combined with the non-photostimulatory green light (29 lx); and green group, photostimulated with the green light combined with the non-photostimulatory red light. Data are presented as mean ± standard error of the mean (*N* = 45). Values with different letters are significantly different (*p* ≤ 0.05) (Mobarkey et al., 2010).

In this trial, we tested our hypothesis on broiler breeder hens with a few technological upgrades by switching to LED devices that provided a similar illumination environment to that described in the first initial trial. Before the study, white light intensity and brightness were measured using a LI-COR light meter (LI-COR, Lincoln, NE, United States) under the standard conditions of the chicken house. Intensity levels (0.1 W/m^2^) and brightness (29 x) were used for green and red illumination, respectively. Annual egg production was significantly elevated in the group exposed to long-day red light together with short-day exposure to green light (Figure 2).

**FIGURE 3:**
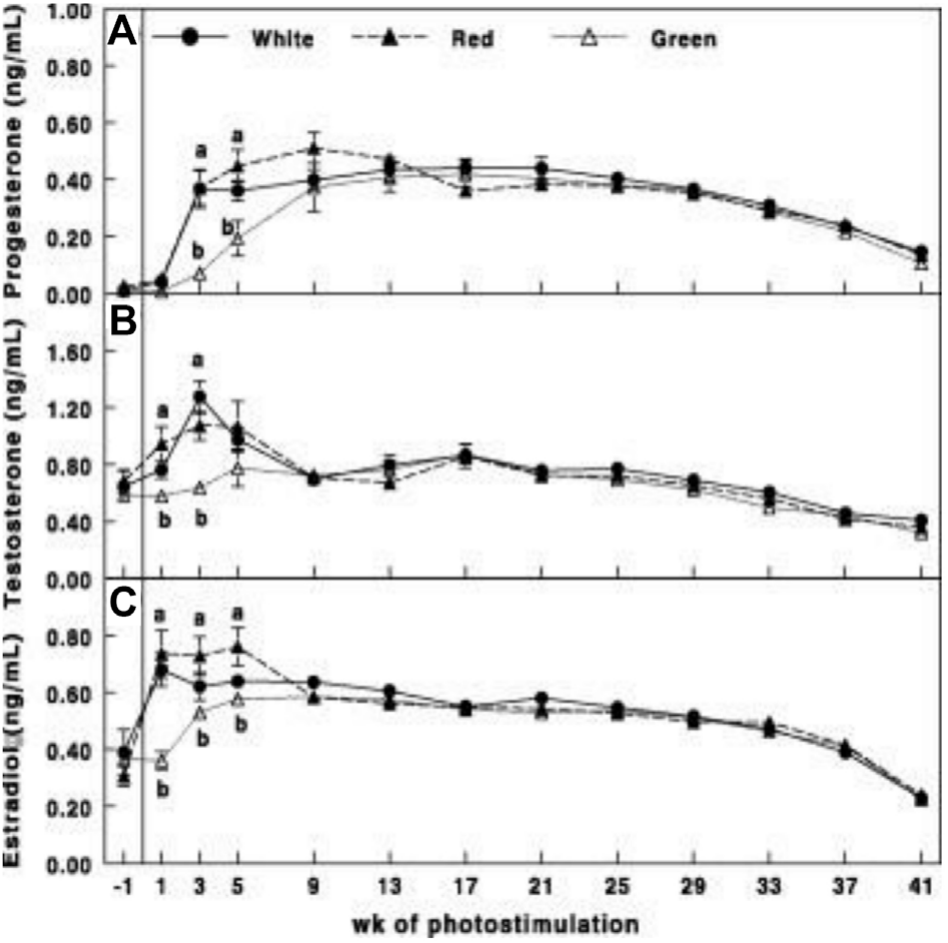
Plasma progesterone **(A)**, testosterone **(B)**, and estradiol **(C)** concentrations of Cobb broiler breeder hens in the control group (29 lux 0.1 W/m^2^); red group, photostimulated with the red light (0.1 W/m^2^) combined with the non-photostimulatory green light (29 lx); and green group, photostimulated with the green light combined with the non-photostimulatory red light. Plasma steroid concentrations were determined by enzyme-linked immunosorbent assay. Data are presented as mean ± standard error of the mean (*N* = 45). Values with different letters are significantly different (*p* ≤ 0.05) (Mobarkey et al., 2010).

**FIGURE 4:**
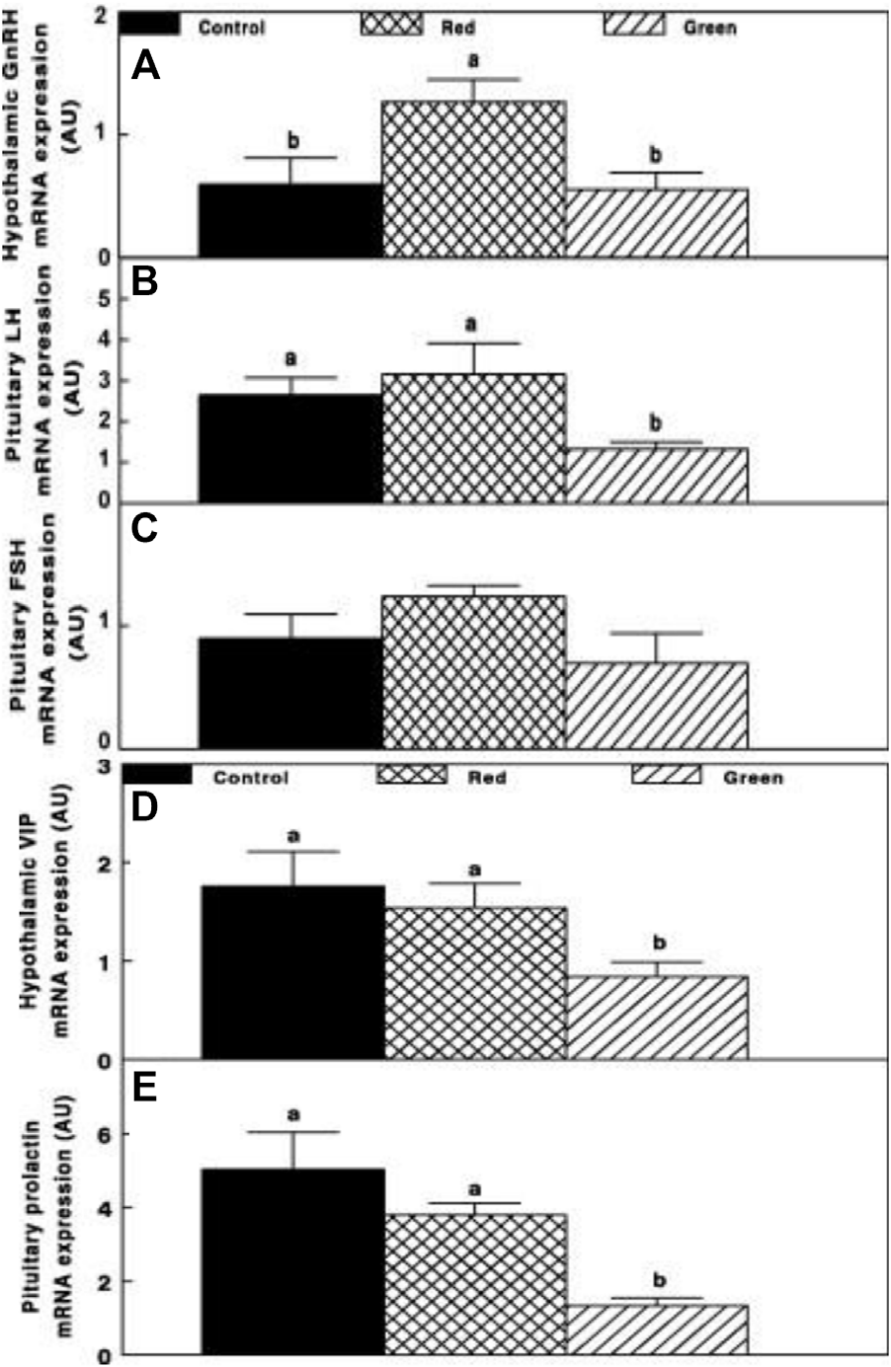
Chicken GnRH **(A)**, LH **(B)**, FSH **(C)**, hypothalamic vasoactive intestinal peptide (VIP) **(D)**, and pituitary prolactin **(E)** mRNA expression of the control group (29 lx, 0.1 W/m^2^); red group, photostimulated with the red light (0.1 W/m^2^) combined with the non-photostimulatory green light (29 lx); and green group, photostimulated with the green light combined with the non-photostimulatory red light. Expression of gonadotropin-releasing hormone (GnRH), luteinizing hormone (LH), and follicle-stimulating hormone (FSH) was determined by semiquantitative PCR. Abbreviation: A.U., arbitrary units. Data are presented as mean ± standard error of the mean (*n* = 4). Values with different letters are significantly different (*p* ≤ 0.05) (Mobarkey et al., 2010).

Gonadal axis activity is elevated in the red group compared to all other illuminated groups. This was manifested by the elevation of gonadal steroids at the initiation of egg production (Figure 3) and hypothalamic GnRH-I, pituitary LH, and FSH mRNA gene expression (Figures 4A–C).

Relative photostimulation of retinal and extraretinal photoreceptors also affected the lactotropic axis. Hypothalamic vasoactive intestinal peptide (VIP) mRNA expression was reduced in retinal photostimulated hens (green group, Figure 4D) and was correlated with decreased prolactin mRNA expression (*p* ≤ 0.05; Figure 4E).

**FIGURE 5:**
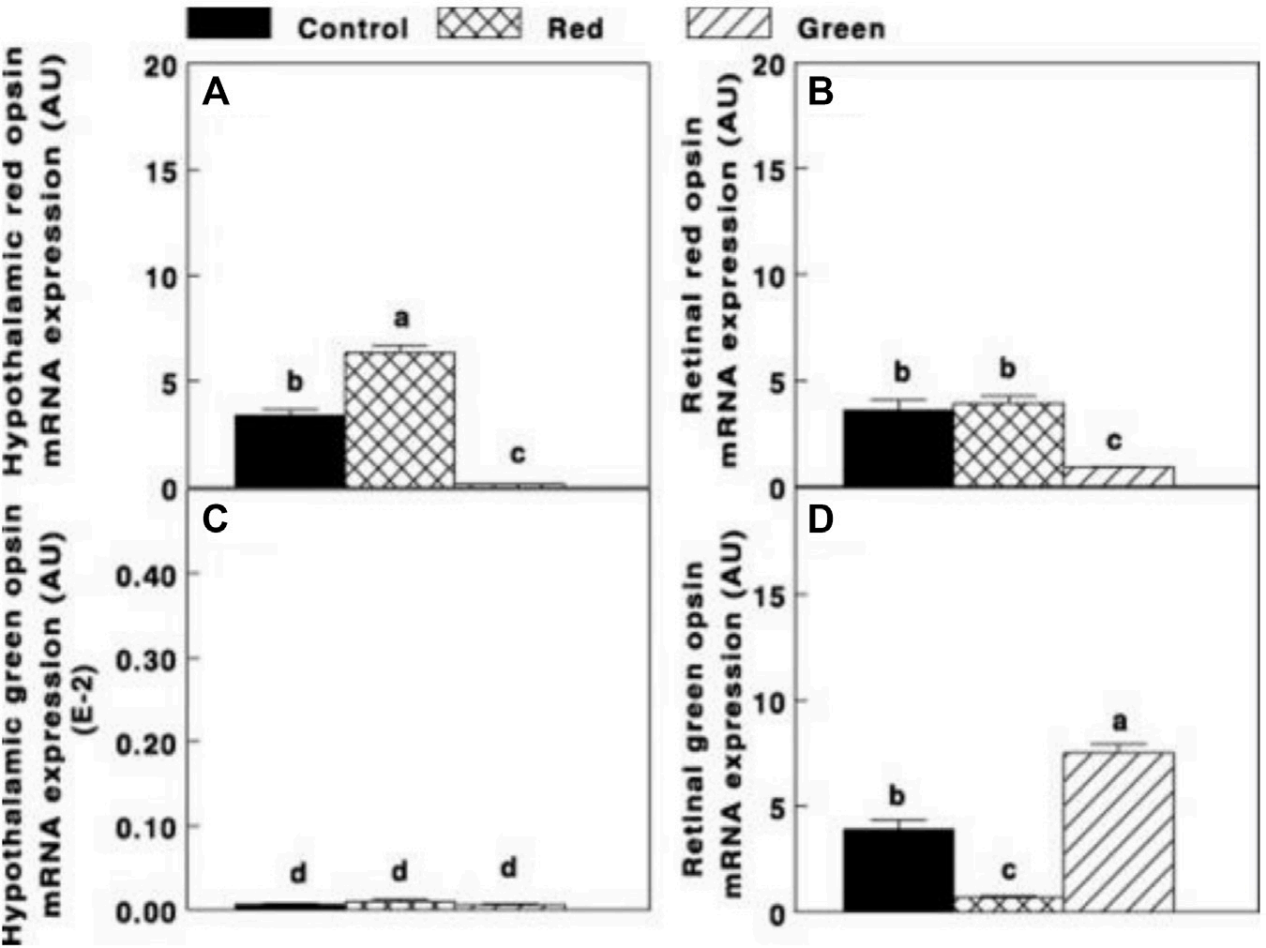
Hypothalamic and retinal red opsin mRNA expression [**(A,B)**, respectively] and hypothalamic and retinal green opsin [**(C,D)**, respectively] of the control group (29 lx, 0.1 W/m^2^); red group, photostimulated with the red light (0.1 W/m^2^) combined with the non-photostimulatory green light (29 lx); and green group, photostimulated with the green light combined with the non-photostimulatory red light. Data are presented as mean ± standard error of the mean (*n* = 5). Values with different letters are significantly different (*p* ≤ 0.05) (Mobarkey et al., 2010).

In addition, activating the brain (ERPR) (red group) significantly elevated (*p* ≤ 0.05) hypothalamic red opsin mRNA expression (Figure 5A). In addition, the expression of red opsin was also observed in the retina (Figure 5B). Selective retinal photoreceptor photostimulation in the green group significantly elevated retinal green opsin (Figure 5C), whereas the expression of green opsin in the hypothalamus was very low in all groups (Figure 5D).

Similar to our previous study conducted on turkeys, the activation of ERPR combined with non-photostimulatory conditions to the retinal photoreceptors of broiler breeder hens significantly elevated gonadal axis activity. A unique finding was observed in the hypothalamus. Photostimulation of the ERPR significantly elevated mRNA gene expression of red opsin, suggesting that it might be related to the elevation of GnRH-I mRNA gene expression of increased cumulative egg production (9.87%, compared to the control group). Furthermore, we suggest a possible direct connection between the hypothalamic ERPR and GnRH synapses (Mobarkey et al., 2010).

The debilitating effect of green photostimulation on reproduction was studied in a separate experiment. Understanding the mechanism(s) of the adverse effect of retinal photostimulation on reproduction was the main objective of the next study presented in this paper. Two target candidates were studied: the lactotropic axis and the serotonergic axis.

Serotonin, which is synthesized in the retina (Dawson and Goldsmith, 1997; Cho et al., 1998) and in the hypothalamus (Dunn and Sharp, 1999) during the day (Dawson and Goldsmith, 1997; Cho et al., 1998; Péczely and Kovács, 2000), has been reported to inhibit avian reproduction (Proudman and Opel, 1981; Bacon and Long, 1996; El Halawani et al., 1996). Elevation of serotonin levels directly inhibits GnRH synthesis (Kikuchi et al., 1998) and LH secretion (Proudman and Opel, 1981; El Halawani et al., 1996). In addition, deactivation of the serotonergic axis generally elevates gonadotropin secretion, followed by gonadal development (El Halawani et al., 1996). VIP’s synthesis and release are controlled by serotonin (Wong et al., 1991; You et al., 1995; Kang et al., 2006), and levels are changed every 24 h (increase during the day and reduce at night) (Menaker and Keatts, 1968). Furthermore, VIP is considered to be a major prolactin-releasing factor (Menaker et al., 1970), and high plasma concentrations of prolactin inhibit reproduction (Benoit and Ott, 1944). Photostimulation increases hypothalamic VIP mRNA content (Homma et al., 1977) and secretion (Foster and Hankins, 2002), which increases prolactin synthesis and secretion (Berson, 2003).

In an experiment published by Mobarkey et al. (2013), broiler breeder hens were photostimulated with the green light while maintaining red light under non-photostimulatory conditions. Parallel to photostimulation, birds were either vaccinated against VIP or orally treated with parachlorophenylalanine (PCPA), which blocks serotonin synthesis by inhibiting tryptophan hydroxylase (an enzyme involved in the pathway of serotonin synthesis) (Silver et al., 1988).

**FIGURE 6:**
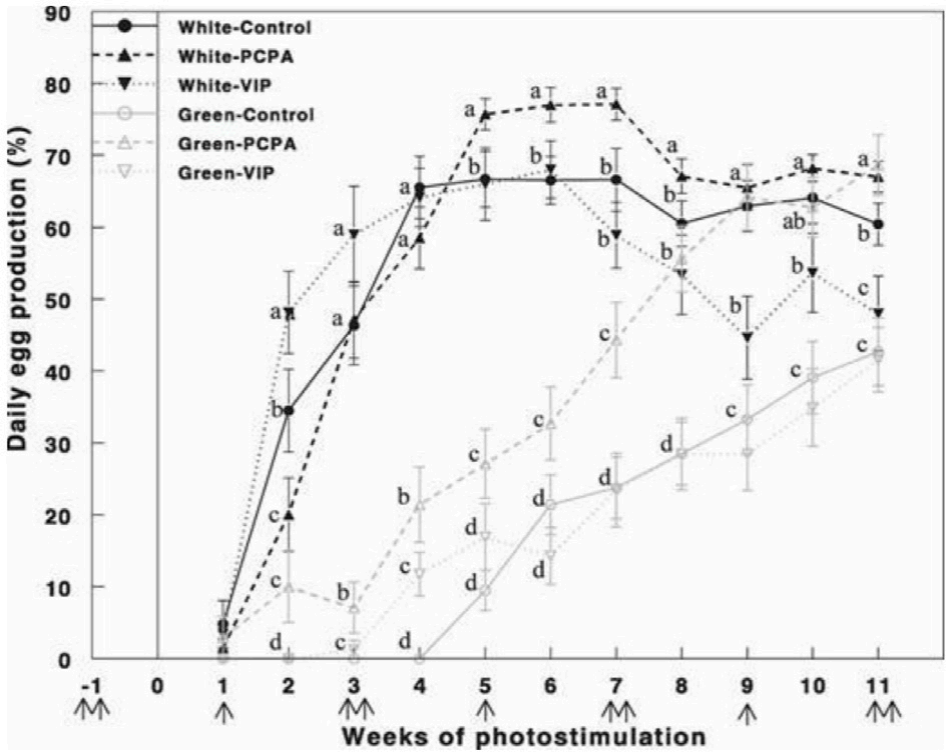
Egg production (%) of Cobb broiler breeder hens reared under photostimulatory white light (29 lx, 0.1 W/m^2^ White) or green light (29 lx) combined with the non-photostimulatory red light (0.1 W/m^2^) (Green). Hens were treated with PCPA, actively immunized against VIP, or left untreated (control). A single arrow indicates PCPA treatment, and double arrows indicate the timing of VIP immunization or PCPA treatment. Data are means ± SEM (*n* = 15). Values with different letters are significantly different (Mobarkey et al., 2013).

Retinal photostimulation of ERPR by the green light under the non-photostimulatory condition by exposing the birds to a short day of red light caused a significant delay in the onset of egg production (Figure 6). Hens that were photostimulated with the green light and treated with PCPA showed improved (*p* < 0.05) egg production compared with that of the green-control and green-VIP groups. Egg production of the green-PCPA group did not differ from that of the white-control, white-PCPA, and white-VIP groups that were photostimulated for 8 weeks. Active immunization against VIP had no effect on egg production under either the white light or green light.

**FIGURE 7:**
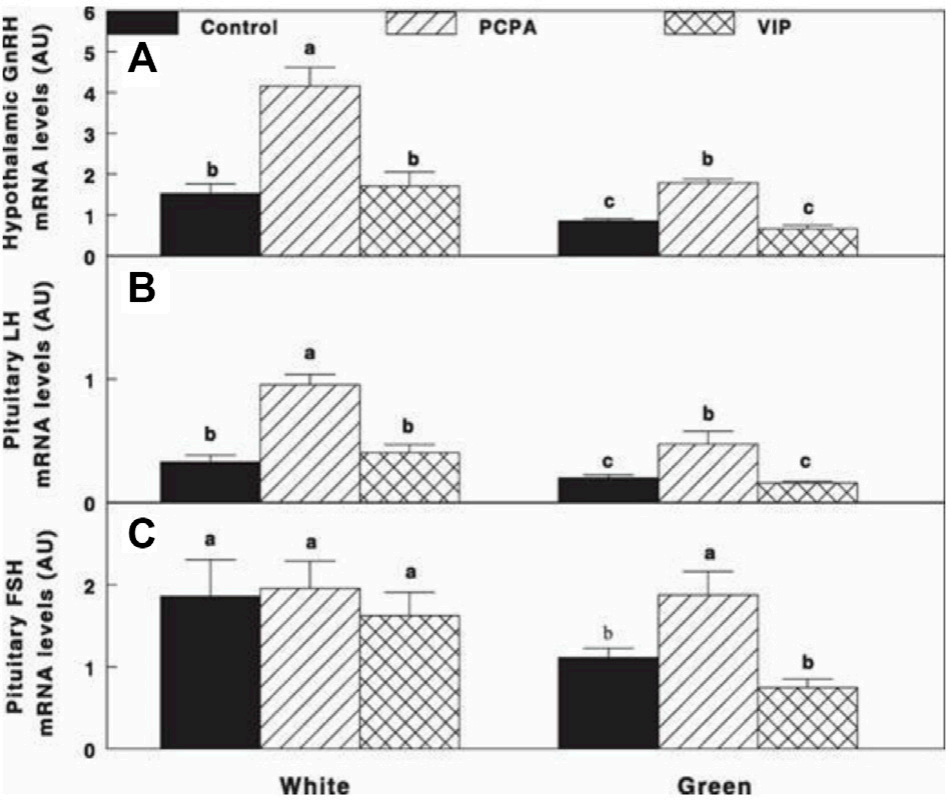
Expression of GnRH-I **(A)**, LH-β **(B)**, and FSH-β **(C)** mRNA of Cobb broiler breeder hens reared under the photostimulatory white light (29 lx, 0.1 W/m^2^ White) or green light (29 lx) combined with the non-photostimulatory red light (Green). Hens were treated with PCPA, actively immunized against VIP, or left untreated (Control). Values with different letters are significantly different (*p* < 0.05) (Mobarkey et al., 2013).

Retinal activation of ERPR with the green light under a non-photostimulatory condition by a short day of red light significantly reduced gonadal axis activity (reduction in GnRH-I, LH-β, and FSH-β mRNA expression) (Figures 7A–C, respectively); PCPA treatment significantly elevated mRNA gene expression of GnRH-I and LH-β to similar levels of the white-control group. Active immunization against VIP had no effect on mRNA gene expression of the aforementioned components of the gonadal axis also in the white-control or green-photostimulated groups.

**FIGURE 8:**
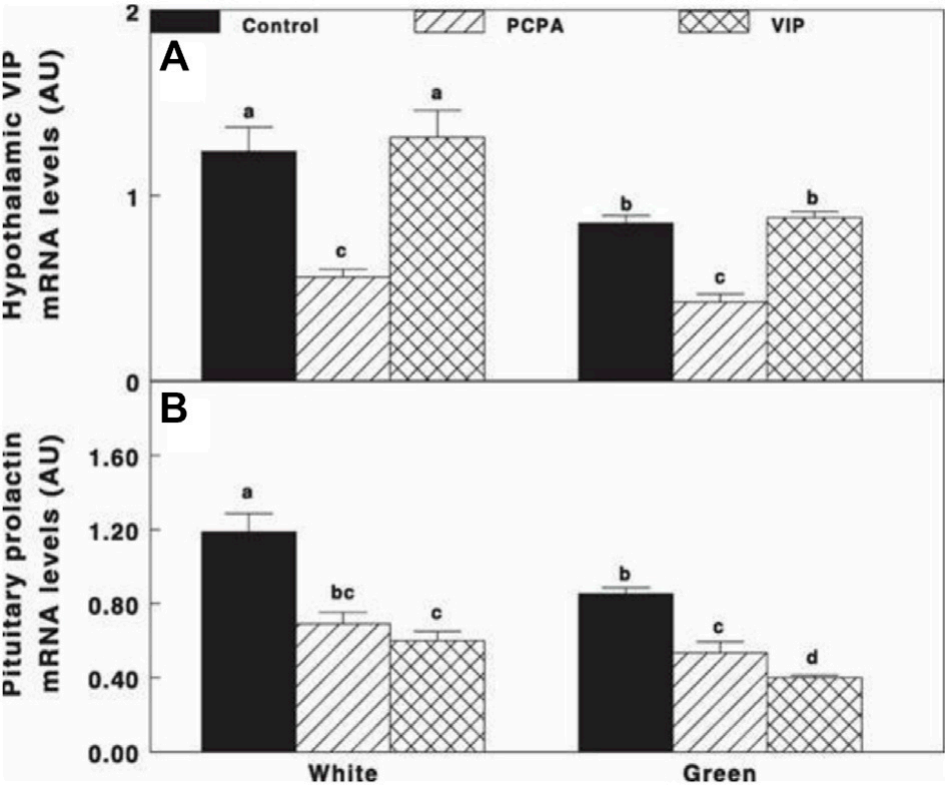
VIP **(A)** and prolactin **(B)** mRNA gene expression of Cobb broiler breeder hens reared under the photostimulatory white light (29 lx, 0.1 W/m2 white) or green light (29 lx) combined with the non-photostimulatory red light (green). Hens were treated with PCPA, actively immunized against VIP, or left untreated (control). Data are means ± SEM (*n* = 4). Values with different letters are significantly different (*p* < 0.05) (Mobarkey et al., 2013).

Oral administration of PCPA significantly reduced VIP and prolactin mRNA gene expression under both white light and green light (Figures 8A,B), followed by significant decrease in prolactin mRNA gene expression without any effect on VIP mRNA gene expression that was similar to that of the control groups of each treatment light.

**FIGURE 9:**
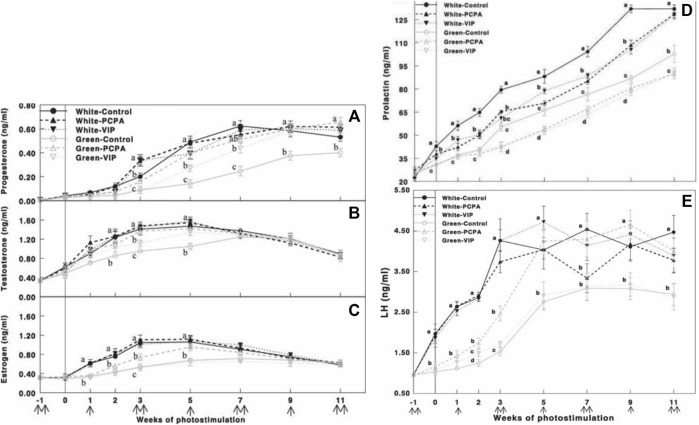
Plasma progesterone **(A)**, testosterone **(B)**, estrogen **(C)**, prolactin **(D)**, and LH **(E)** concentrations determined by ELISA of Cobb broiler breeder hens reared under the photostimulatory white light (29 lx, 0.1 W/m^2^ white) or green light (29 lx) combined with the non-photostimulatory red light (green). Hens were treated with PCPA, actively immunized against VIP, or left untreated (control). Data are means ± SEM (*n* = 15). Values with different letters are significantly different (*p* < 0.05) (Mobarkey et al., 2013).

PCPA treatment of green light-photostimulated birds significantly elevated plasma LH compared to green-control hens and green-VIP groups (Figure 9E). Plasma LH levels in the green light PCPA-treated group were elevated at 5 weeks of photostimulation, with no significant difference in the white-control groups.

PCPA and VIP treatments of all light groups (white and green light) significantly reduced plasma prolactin levels (Figure 9D). Similarly, the green light-photostimulated group showed lower gonadal steroid levels. Oral administration of PCPA elevated plasma gonadal steroids, thus overcoming the debilitating effect of photostimulation with the green light. Furthermore, active immunization against VIP also significantly elevated plasma progesterone and testosterone levels; however, this elevation was smaller than that in the PCPA treatment group. Active immunization against VIP had no effect on plasma estrogen concentrations (Figures 9A‐C).

The mechanism(s) by which retinal photostimulation inhibits reproduction is far from clear. Several speculations can be made: gonadotropin-inhibitory hormone (GnIH) was found to inhibit gonadotropin release from the anterior pituitary of chickens (Ohta and Homma, 1987; Meddle and Follett, 1997). Further studies are needed in order to verify whether retinal photostimulation debilitates reproductive activities by elevating GnIH. We suggest that serotonin plays a pivotal role and should be investigated in relation to retinal inhibition of reproduction. This suggestion is based on several studies indicating that serotonin is synthesized in the retina (Millam et al., 2003; Yasuo et al., 2003) and in the hypothalamus (Harrison, 1972) that inhibits avian reproduction (El Halawani et al., 1983; Hall et al., 1986). Levels of inhibition can be shown at the hypothalamic level by GnRH inhibition (El Halawani et al., 1995) and LH secretion (Hall et al., 1986). Conversely, a blockade of the serotonergic system generally stimulates gonadotropin secretion and enhances gonadal development (Ubuka et al., 2005).

The effects of targeted wavelength illumination on reproductive activities have been much less studied in roosters than in hens. Early studies presented the effect of wavelength stimulation on sexual maturation of several male birds, in which long wavelength stimulation has been shown to accelerate maturation in roosters (Johnson et al., 1982), ducks (Benoit et al., 1950), and quails (Woodard et al., 1969). In addition, roosters that were photostimulated with the white or red light for 6 h had lower spermatogenesis and GnRH levels and lower testis weight than roosters that were photostimulated for 14 h. A similar study using green or blue wavelengths did not have the same effect (Casey et al., 1969). Male semen quality (concentration, viability, and motility) is highly correlated with fertility. Lower levels of semen quality can result in subfertile rooster and great economic losses (McDaniel et al., 1998). As in females, we hypothesized that targeted illumination in roosters might elevate fertility and reproductive performances.

## The effect of targeted photostimulation on broiler breeder males

In a newly published study, differential targeted photostimulation was tested on broiler breeder roosters. Similar to the illumination protocol that was used on broiler breeder hens, ERPR photostimulation was applied by red-light illumination (14 h) while maintaining non-photostimulatory conditions for the retinal photoreceptors (illumination for 6 h with either blue or green light), as shown in Table 1.

**TABLE 1:** Lighting regime and treatments.

Treatment	White light	Blue light	Red light	Green light	Total hours
Control group	0,700–2,100 h	—	—	—	14
Blue–red group	—	0,700–2,100 h	0,700–1,300 h	—	14
Red–blue group	—	0,700–1,300 h	0,700–2,100 h	—	14
Green–red group	—	—	0,700–1,300 h	0,700–2,100 h	14
Red–green group	—	—	0,700–2,100 h	0,700–1,300 h	14

**FIGURE 10:**
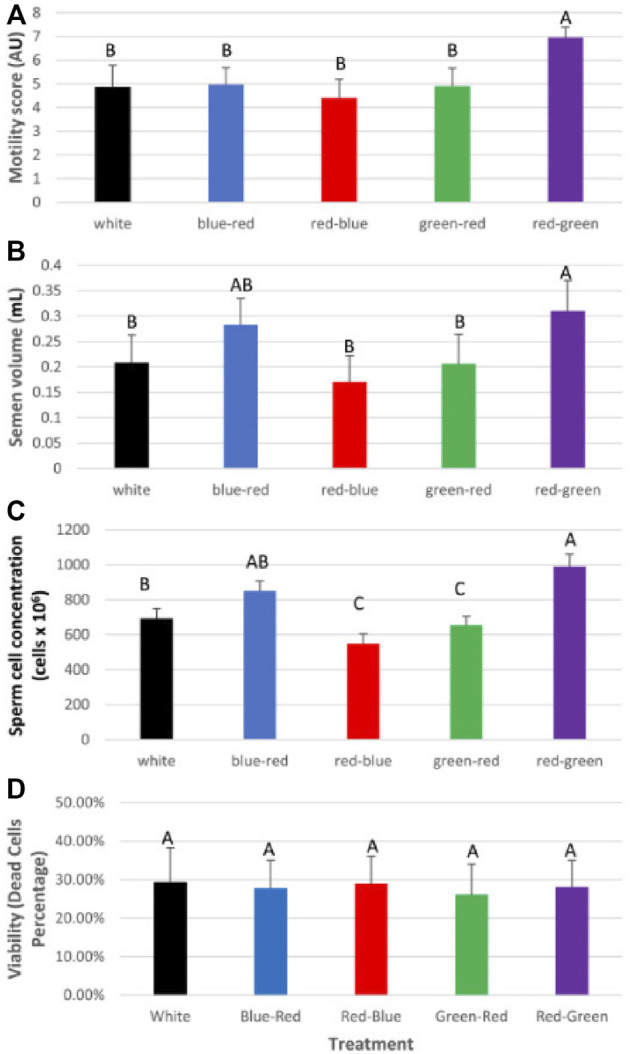
Semen analysis (average) for the experimental duration of broiler breeder roosters (Ross) exposed to: 14 h white light, control (white); blue light for 14 h combined with 6 h red light (blue–red); red light for 14 h with 6 h blue light (red–blue); green light for 14 h with 6 h red light (green–red); and red light for 14 h with 6 h green light (red–green). **(A)** Motility rate in all groups. **(B)** Semen volume. **(C)** Concentration of sperm cells per ejaculate. Data are presented as average ±standard error. Levels with different letters are significantly different (*p* ≤ 0.05). **(D)** Viability of sperm cells. Data are presented as average ± standard error. Levels with different letters are significantly different (*p* ≤ 0.05) (Bartman et al., 2021).

**FIGURE 11:**
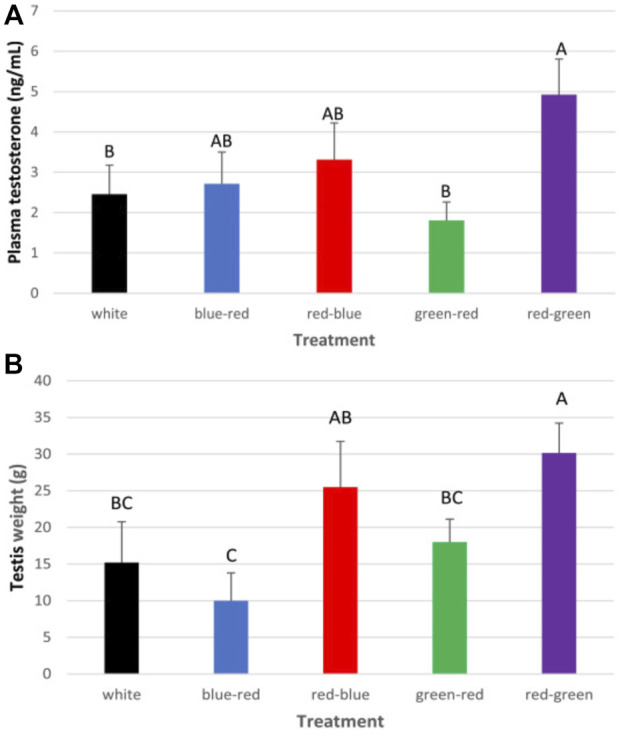
Cumulative plasma testosterone levels **(A)** and weight of testes **(B)** of all roosters after exposure to: 14 h white light, control (white); blue light for 14 h combined with 6 h red light (blue–red); red light for 14 h with 6 h blue light (red–blue); green light for 14 h with 6 h red light (green–red); and red light for 14 h with 6 h green light (red–green). Data are presented as average ± standard error. Levels with different letters are significantly different (*p* ≤ 0.05) (Bartman et al., 2021).

After roosters were photostimulated (as previously described for broiler breeder hens), individual semen quality analysis (semen volume, motility, sperm cell concentration/ml, concentration/ejaculate, and viability) was conducted weekly until the end of the experiment at 65 weeks of age. Plasma samples were tested monthly for prolactin, estradiol, progesterone, and testosterone levels. At 65 weeks of age, all roosters were euthanized, and selected tissues were collected for mRNA gene expression analysis. The results of this study show that semen quality parameters are significantly elevated in red–green group (long day red light combined with short day green light) compared to the control white light-photostimulated group (Figure 10). Furthermore, both long-day photostimulated groups (red–green and red–blue) had higher testis weight at 65 weeks of age (Figure 11B). Moreover, plasma testosterone levels were significantly elevated in the red–green group (average 4.99 ng/ml), compared to those of all other treatment and control groups that averaged 3.04 ng/ml (Figure 11A). The difference was significant compared to the green–red treatment and control group.

**FIGURE 12:**
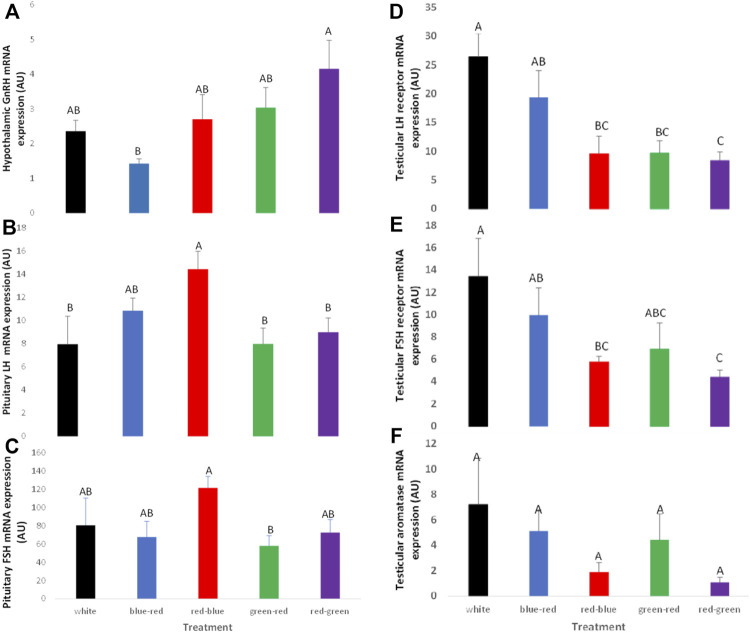
Expression of GnRH-I **(A)**, LH-β **(B)**, FSH-β **(C)**, testicular LH receptors **(D)**, FSH receptors **(E)**, and aromatase **(F)** mRNA of broiler breeder roosters (Ross) exposed to: 14 h white light, control (white); blue light for 14 h combined with 6 h red light (blue–red); red light for 14 h with 6 h blue light (red–blue); green light for 14 h with 6 h red light (green–red); red light for 14 h with 6 h green light (red–green). Data are presented as average ± standard error. Levels with different letters are significantly different (*p* ≤ 0.05) (Bartman et al., 2021).

Similarly to broiler breeder hens, acceleration of gonadal axis activity was detected in the red–green treatment group manifested by higher hypothalamic GnRH mRNA gene expression levels and pituitary LH and FSH mRNA levels (Figures 12A–C, respectively). In addition, LH and FSH receptor mRNA gene expression in the testes was lower in the red–green group than that in all other treatment groups, including the control group (Figures 12D,E). Aromatase mRNA levels in the testes were the lowest in the red–green treatment group (Figure 12F). Although the difference was not significant, the long-day red treatments resulted in lower expression of this gene than all other treatments and most of all to the control, which showed the highest level of expression (Bartman et al., 2021).

## Discussion

Temperate zone birds, including broilers and turkeys, are photoperiodic, with increasing day length stimulating sexual activity, which is caused by long wavelengths of the spectrum (Benoit, 1964; Woodard et al., 1969). Photoperiodic activation of the reproduction state is mediated by ERPR (Benoit and Ott, 1944; Menaker et al., 1970). The location of the ERPR associated to photoperiodic stimulation of reproduction is the medio-basal region of the hypothalamus (Homma et al., 1977; Foster and Hankins, 2002). Unfortunately, little is known about extraretinal photoreceptors, and consequently, we know very little about their sensory physiology or molecular biology. Several opsins have been characterized both in cells and tissues beyond the traditionally accepted retinal photoreceptors (the rods and cones) in several vertebrate species (Berson, 2003; Van Gelder, 2003; Silver et al., 1988; Zaguri et al., 2020; Mobarkey et al., 2010). Immunoreactivity of opsin neurons were found in the quail and duck medio-basal hypothalamus (MBH) (Ohta et al., 1984). Furthermore, electrical stimulation of this region resulted LH secretion and gonadal growth (Sharp and Follett, 1969; Konishi et al., 1987). In addition, MBH lesions block the photo-induced release of LH and testicular growth (Davies and Follett, 1975; Ohta and Homma, 1987; Meddle and Follett, 1997). Quail and turkey neuronal activation, manifested by fos-like protein expression, occurs in the MBH and is associated with photoperiodically driven LH rise (Millam et al., 2003; Yasuo et al., 2003). All evidence points to the MBH as a pivotal site for circadian measurement of day length (Harrison, 1972).

**FIGURE 13:**
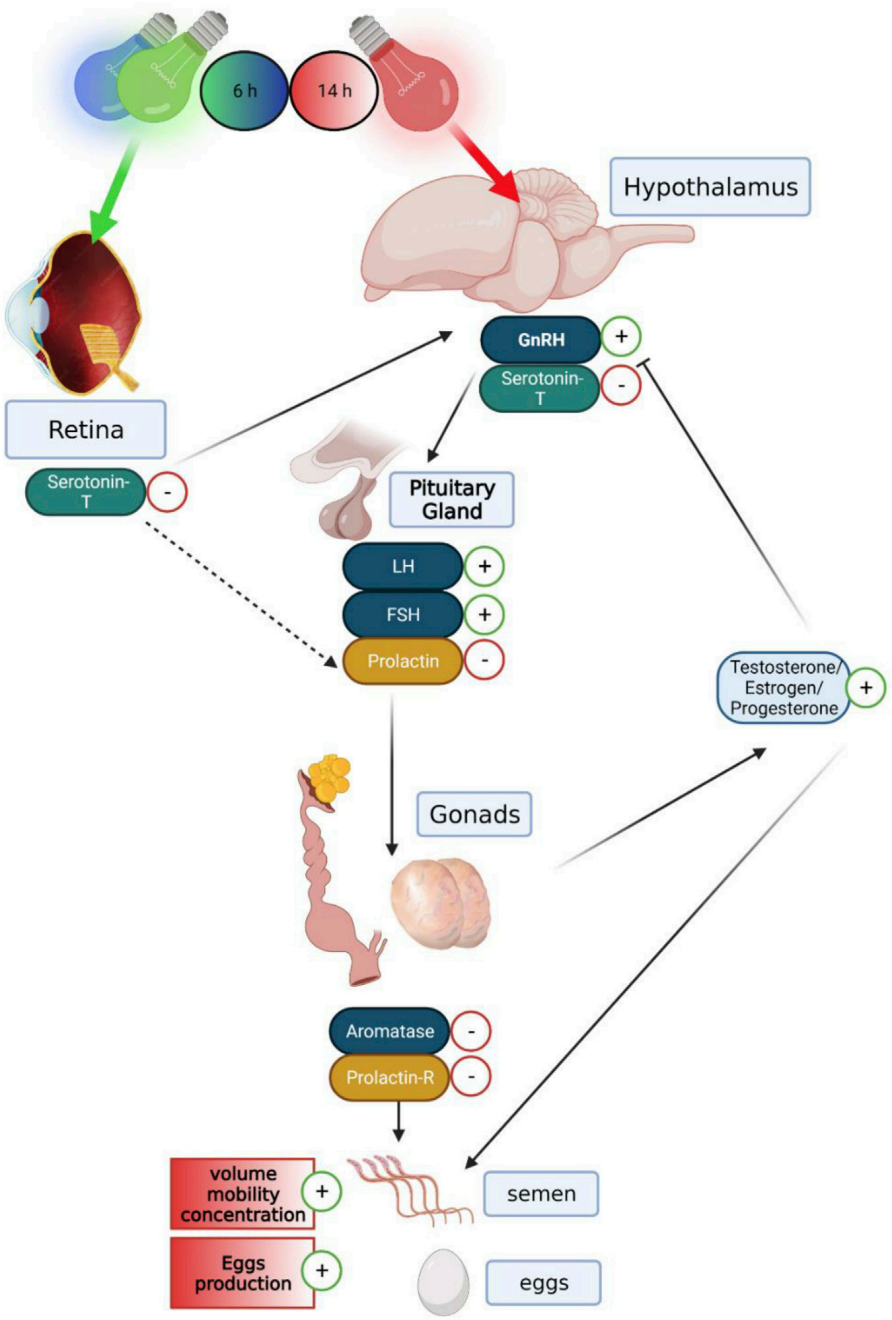
Graphical illustration demonstrating the hormonal and genomic changes of the gonadal, serotonergic, and lactotropic axes in the hypothalamus–pituitary–gonadal pathway, in both breeding broiler males and females: a long day of red illumination combined with a short day of green or blue illumination resulting in elevated expression levels of GnRH mRNA and reduced levels of serotonin transporter in the hypothalamus. Furthermore, a lower level of serotonin transporter expression levels in the retina can be observed. This causes raised levels of LH and FSH gene expression and lower gene expression of prolactin in the pituitary gland. This follows a reduction in aromatase and prolactin receptor mRNA expression in the reproductive systems, thus causing elevation in testosterone levels in the plasma and higher semen quality in males and elevation in estrogen and progesterone in the plasma and larger egg production in females. Created with BioRender.com.

The retina is the most obvious photoreceptive tissue that is activated by the green spectrum (Prescott and Wathes, 1999; Lewis and Morris, 2000). There are indications that the activation of retinal photoreceptors by visible radiation is inhibitory to reproduction (Homma et al., 1972; Siopes and Wo, 1980). Orbital enucleation increased egg production in chickens (Ubuka et al., 2005), and orbital enucleation combined with pinealectomy decreased the hypothalamic concentration of gonadotropin-inhibitory factor (GnIH) mRNA and its peptide (Underwood et al., 1990). These findings, taken together with our results, suggest of two light pathways that regulate reproduction in birds, as shown in Figure 13: a stimulatory pathway mediated by hypothalamic photoreceptors, which are activated by the red spectrum/630 nm wavelength and an inhibitory retinal-hypothalamic pathway activated by the green spectrum/525 nm wavelength. The functional significance of the interaction between the two hypothesized pathways in the regulation of the avian reproductive cycle is currently unclear. The possibility remains that the retinal pathway may be of importance at the termination of egg-laying activity, as in the case of the onset of photorefractoriness. The mechanism underlying photorefractoriness is unknown, but there are indications that the retinal-hypothalamic pathway, which involves the melatonin system (Underwood et al., 1984; Guyomarc’h et al., 2001), may be involved in the inhibition of the avian reproductive neuroendocrine system and the termination of sexual and egg-laying activities (Rozenboim et al., 2002). More recently, melatonin injection has been shown to increase hypothalamic levels of GnIH mRNA and its peptide (Underwood et al., 1990).

Long wavelengths (red light) penetrate the skull and tissues and stimulate ERPR—activating the gonadal axis (Oishi and Lauber, 1973; Benoit, 1978; Mobarkey et al., 2010). Shorter wavelengths, (green–yellow lights) mainly activating the retina, stimulate the secretion of gonadotropin-inhibitory hormone (GnIH), followed by reproduction inhibition (Benoit and Assenmacher, 1966; Mobarkey et al., 2013; Bédécarrats, 2015). Selective photostimulation of different photoreceptor sites can be used as an environmental tool for acceleration of reproductive activities in domestic birds.

Several endocrine axes are involved in reproductive activities of domestic birds, and the most pronounced ones are the gonadotropic axis and the lactotropic/serotonergic axis, both known to be activated by photostimulation. The gonadotropic axis activation by photostimulation has been well characterized (Sharp et al., 1998; Saldanha et al., 2001), whereas the lactotropic and serotonergic axes are known to deactivate reproduction **(** Avital- Cohen et al., 2012). The mechanisms through which photic cues are transduced to neuroendocrine effector neurons remain unknown. There is still much to discover about the connection between the brain ERPR and the reproductive axis. Previous studies have shown that brain photoreceptors communicate directly with the GnRH neurons that stimulate the activation of reproduction (Saldanha et al., 2001; Scanes and Dridi, 2021). Another possible connection is with vasoactive intestinal peptide (VIP) cells, which colocalize with all opsin-expressing cells in birds. Within the opsin system, VIP could potentially regulate reproduction through synaptic interactions all along the trajectory of its axons through the lateral septum and hypothalamus (Hof et al., 1991; Saldanha et al., 2001). More importantly, we previously demonstrated that complementary treatment with PRL in old breeder roosters vaccinated against VIP reactivated the gonadal axis activity and improved sperm quality (Avital- Cohen et al., 2012). Thus, the decline in VIP and PRL gene expression in the green–red group might explain the damage caused to LH gene expression and reproductive performance.

Elevation in GnIH mRNA gene expression was shown in birds subjected to long-day green light combined with short-day red light (green–red group) (Zaguri et al., 2020). Since GnIH inhibits the synthesis and secretion of gonadotropins LH and FSH in domestic fowl (Ciccone et al., 2004) and disrupts gonadal development and activity in quail (Ubuka et al., 2006), we suggest the involvement of this hormone as a deactivator of reproduction.
